# Diel Oscillation of Microbial Gene Transcripts Declines With Depth in Oligotrophic Ocean Waters

**DOI:** 10.3389/fmicb.2019.02191

**Published:** 2019-09-24

**Authors:** Alice Vislova, Oscar A. Sosa, John M. Eppley, Anna E. Romano, Edward F. DeLong

**Affiliations:** Daniel K. Inouye Center for Microbial Oceanography: Research and Education, University of Hawaii, Honolulu, HI, United States

**Keywords:** diel, oceanography, oligotrophic, bacterioplankton, phytoplankton, transcriptome

## Abstract

Diel oscillations in primary and secondary production, growth, metabolic activity, and gene expression commonly occur in marine microbial communities in ocean surface waters. Diel periodicity of gene transcription has been demonstrated in photoautotrophic and heterotrophic microbes in both coastal and open ocean environments. To better define the spatiotemporal distribution and patterns of these daily oscillations, we investigated how diel periodicity in gene transcripts changed with depth from the surface waters to the upper mesopelagic. We postulated that diel oscillation of transcript abundances would diminish at greater depths across the collective microbial community due to decreasing light availability. The results showed that the number and total proportion of gene transcripts and taxa exhibiting diel periodicity were greatest in the shallow sunlit mixed layer, diminished rapidly with increasing depth to the base of the euphotic zone, and could not be detected in the mesopelagic. The results confirmed an overall decrease in microbial diel transcript oscillation with depth through the euphotic zone and suggested a relationship between abundance of diel oscillating transcripts and the daily integrated light exposure experienced by planktonic microbes in the water column. Local dissolved macronutrient concentration also appeared to influence the diel transcriptional patterns of specific microbial genes. The diminishing diel transcript oscillations found at increasing depths suggest that diel patterns of other microbial processes and interactions may likewise be attenuated at depth.

## Introduction

Marine microbes dominate biomass, biodiversity, and metabolism in the oceans and form the foundation of marine food webs and major biogeochemical cycles on Earth (Azam and Malfatti, 2007; Falkowski et al., 2008). Marine phytoplankton are responsible for approximately one-half of global primary production, about 75% of which takes place in the vast oligotrophic gyres (Karl and Church, 2014). The largest gyre, the North Pacific Subtropical Gyre (NPSG), spans 2×10^7^ km^2^ from 15°N to 35°N and from 135°E to 135°W (Sverdrup et al., 1946). Wind-driven mixing and seasonally stable temperatures in the NPSG produce a uniform, warm (>24°C), low-density surface mixed layer ranging from 20 to 100 m, which is separated year-round from the rest of the density-stratified water column (Karl and Church, 2017). The microbial community throughout the upper mixed layer experiences uniform low-nutrient conditions and the delivery of equal amounts of time-integrated solar irradiation, when overturning within the mixed layer. Although in eutrophic coastal regions, strong macronutrient upwelling tends to select for larger phytoplankton species (Tiera et al., 2001; Cotner and Biddanda, 2002), in the NPSG, limited macronutrient supplies from greater depths foster a microbial community dominated by smaller photosynthetic picoplankton (Pomeroy et al., 2007). Below the mixed layer, time-integrated light available for photosynthesis attenuates exponentially in quality and quantity with depth through the euphotic/epipelagic zone (~upper 200 m in the NPSG), the base of which is functionally defined as the depth of 1% of surface irradiation (also considered to be the compensation depth; Karl, 1999).

Driven by direct interaction with the oscillating light energy field, diel periodicity in phytoplankton activity is well documented and regulated by a number of phylogenetically distinct circadian clocks across the tree of life. Circadian clocks are composed of oscillators made up of positive and negative elements forming feedback loops, which continue to regulate periodic gene expression, giving organisms the advantage of being able to prepare for periodic events in advance even in the absence of entraining in the light signal (Bell-Pedersen et al., 2005). Early studies reporting diel oscillations in marine phytoplankton preceded the discovery of picocyanobacteria in the open ocean, and therefore focused on eukaryotic species. These pioneering studies reported diel periodicity in algal cell division (Nelson and Brand, 1979), and photosynthetic capacity and chlorophyll concentrations (Sournia, 1975).

*Prochlorococcus*, the smallest and most abundant photosynthetic organism on Earth and the most abundant oxygenic phototroph in low and mid latitude marine environments (Chisholm et al., 1988), was first noted to behave periodically *via in situ* flow cytometry observations of synchronized growth and replication in wild populations, with DNA replication taking place in the afternoon and cell division at night (Vaulot et al., 1995). Additionally, *Prochlorococcus* populations in deeper waters appeared to divide earlier in the day, than did those in surface waters (Vaulot et al., 1995). A number of genetic and physiological studies further characterized diel synchronicity across diverse *Prochlorococcus* cellular processes. *Prochlorococcus* periodicity in substrate uptake (Mary et al., 2008), pigment ratios, and optical properties (spectral attenuation and absorption coefficients) was reported in cultured isolates (Claustre et al., 2002; Bruyant et al., 2005). Transcriptomic analysis of *Prochlorococcus* cultures reported around 90% periodicity of expressed genes, including carbon fixation expression peaking just before dawn, photosynthesis genes with transcript maxima near midday, and transcript counts of genes associated with DNA replication, cell division, carbon catabolism and transporters, especially ammonium transporters, peaking in the evening (Zinser et al., 2009; Waldbauer et al., 2012). The central clock mechanism of cyanobacteria was first discovered in *Synechococcus*, and is made up of three core oscillator genes – *kaiA*, *kaiB*, and *kaiC.* The clock of *Prochlorococcus* strains has a truncated or completely absent *kaiA* gene (Axmann et al., 2009), resulting in a broken clock that functions as a 24-h timer, requiring daily light entrainment in contrast to the function of a complete circadian clock, which continues to oscillate for multiple days under constant light conditions (Holtzendorff et al., 2008).

While transcriptionally driven circadian clocks are widespread across the tree of life, they have not been demonstrated to exist in heterotrophic bacterial species, including heterotrophic bacterioplankton. Nevertheless, diel periodicity in bacterioplankton assemblages and within specific phylogenetic groups has been reported. Heterotrophic bacterioplankton periodicity was first observed in bulk metrics of biomass and metabolic activities peaking during the day, with some delay, after phytoplankton activity (Burney et al., 1982; Johnson et al., 1983; Fuhrman et al., 1985). Metatranscriptome sequencing later showed that some of the most abundant heterotrophic picoplankton (including *Pelagibacter*, *Puniceispirillum*, SAR86, Marine Group II euryarchaeota, and other bacterial groups) exhibited periodicity in transcript abundances for genes associated with translation, transcription, oxidative phosphorylation (Ottesen et al., 2014; Aylward et al., 2015), transporters (Ottesen et al., 2014), and biosynthesis (Poretsky et al., 2009). The mechanisms and drivers of periodicity in heterotrophic bacterioplankton are as yet unknown. One hypothesis is that adjacent steps in catabolism of organic molecules may be temporally partitioned among different metabolic and taxonomic microbial groups, which compete for, as well as provide one another with, dissolved nutrients (McCarren et al., 2010). This tight, temporally-phased metabolic coupling may structure microbial community activities around the diel cycle in surface waters, resulting in widespread diel periodicities in gene expression across diverse microbial taxa and metabolic functions (Ottesen et al., 2014; Aylward et al., 2015). Temporal staggering of peak expression times in similar suites of genes among different taxa was postulated to reflect metabolic cascades that induce periodicity *via* diel pulses of specific extracellular nutrients (Aylward et al., 2015). This hypothesis is consistent with earlier observations of daily fluctuations in labile DOC associated with phytoplankton activity (Meyer-Reil et al., 1979; Burney et al., 1982; Fuhrman and Ferguson, 1986; Leboulanger et al., 1997), and diel oscillations in uptake rates of organic compounds such as carbohydrates (Sieburth et al., 1977; Burney et al., 1982; Johnson et al., 1983), amino acids, (Carlucci et al., 1984), adenine (Winn and Karl, 1986), and nitrogenous substrates (Wheeler et al., 1989).

Although diel periodicity in specific microbial gene transcripts has been reasonably well documented, it has not been extensively investigated below the ocean’s surface layer. Diel periodicity is predicted to be driven by light availability, and therefore is expected to decrease with decreasing light availability below the upper mixed layer. However, how patterns of microbial community diel periodicity change with depth still remains to be determined. Furthermore, transcriptome dynamics may be driven by other factors than light. Depth-stratification of different microbial taxa and processes, as well as depth-variable parameters including light, substrate availability, temperature, and pressure (DeLong et al., 2006; Karl and Church, 2014), may also affect the observed patterns of microbial community diel oscillation.

In this study, we tested the hypothesis that diel patterns in microbial transcriptional activity will diminish with increasing depth, primarily due to light attenuation. We examined depth-dependent changes in diel patterns in transcript abundance, the overall magnitude of diel periodicity, and the taxonomic and functional identity of periodic genes. Two different diel time series depth profiles were conducted during the course of two expeditions in 2014. This allowed us to investigate how different seasons and different upper water column hydrographic conditions might influence patterns of microbial diel transcript periodicity in depth profiles within and below the ocean’s euphotic zone.

## Materials and Methods

### Hydrographic Measurements

Samples and data included in this study were collected during two oceanographic cruises on board the R/V *Kilo Moana*, which were part of the Hawaii Ocean Experiment-Budget of Energy (HOE-BOE) project. Collection of samples for transcriptomic analysis and much of the ancillary data was performed by lowering a frame fitted with a Sea-Bird brand CTD (the conductivity, temperature, and depth sonde) and rosette of Niskin sampling bottles overboard using the ship’s winch and crane. In addition to conductivity (used as a proxy for density) and temperature, the CTD was also equipped to collect vertical profiles of dissolved oxygen and nitrate (the later using a chemical-free, Satlantic brand *In Situ* Ultraviolet Spectrophotometer).

### Solar Irradiation

A Satlantic free-falling optical profiler, also known as the HyperPro, was used to characterize the attenuation of irradiance with depth. The HyperPro was fitted with one up-looking and one down-looking hyperspectral radiometer, as well as chlorophyll, temperature, and conductivity sensors. This instrument also incorporated a ship-mounted surface radiometer. The HyperPro was deployed from the stern through a small block hung from the A-frame. The instrument was hand-lowered and retrieved with assistance from the winch. Surface irradiance was measured continuously throughout both cruises with a PAR sensor.

### Sample Collection

Water for transcriptomic sampling was collected from four depths (25, 75, 125, and 250 m) *via* Niskin bottles mounted on the CTD rosette. Water samples were collected at approximately 4-h intervals for approximately 48 h during each of the two cruises, beginning at 1:00 am on 3/14/2014 during HOE-BOE cruise #1 (KM1409) in March 2014^1^, and 1:00 am on 6/18/2014 during HOE-BOE #2C (KM1413) in June 2014 (See footnote 1). During the March cruise, toward the end of the sampling period, sampling was interrupted due to a storm resulting in a 12-h gap. Storm conditions appear to have deepened the mixed layer during the March cruise. Two liters of water were collected for each transcriptomic sample. Within an hour of their retrieval, seawater samples were passed directly (without prefiltration) through a 0.2 micron Sterivex GV filter (Millipore Sigma, Burlington, MA) by a peristaltic pump. Following filtration, filters were immediately preserved in RNA later and then stored at −80°C. It is important to note that in this study, Lagrangian sampling was not performed, and measurements were taken over less than 2 diurnal cycles.

### RNA and Extraction and Purification

Total RNA extractions were performed by slowly thawing samples on ice and removing preservation solution using a syringe (Frias-Lopez et al., 2008). Then, 350 μl of MirVana denaturing lysis buffer (Ambion, #AM854OG, Austin, TX) was added to the filter unit, capped, and vortexed for 1 min. Next, 350 μl of RNase-free water was added to the filter unit. Lysate was recovered from the filter unit and added to 600 μl of RNase-free water. Prior to purification, lysate was spilt into two aliquots of 675 μl each. Purification and DNase treatment were performed on a Chemagic MSM robotic lab instrument (Perkin Elmer, Waltham, MA) using an RNA Tissue kit (Perkin Elmer, CMG-1212A, Waltham, MA). Total RNA quality was analyzed using a fragment analyzer with the high sensitivity RNA kit from Agilent (DNF-472-0500, Santa Clara, CA), and quantification performed using the Quant-IT Ribogreen RNA kit (Invitrogen, R11490, Carlsbad, CA).

### Metatranscriptome Library Preparation and RNA Sequencing

Total RNA was prepared for sequencing using the ScriptSeq RNA-Seq V2 library preparation kit (Illumina, SSV21124, San Diego, CA) to label cDNA with a unique single-plex barcode. cDNA libraries were quantified using the Quanti-It dsDNA Picogreen kit (Invitrogen, P11496, Carlsbad, CA) with a Victor X3 spectrophotometer, and average cDNA library size was assessed with a Fragment Analyzer using the high sensitivity NGS fragment kit from Agilent (DNF-486-0500, Santa Clara, CA). Metatransciptomic libraries were sequenced on a NextSeq 500 (Illumina, San Diego, CA) (Bennett, 2004) using three mid output V2 reagent kits (Illumina, FC-404-2003) and 1 high output V2 reagent kit (Illumina, FC-404-2003, San Diego, CA) to produce approximately 10 million paired end reads at 150 base pairs length per sample.

### Sequence Analyses and Workflow

Paired end sequences were joined using PandaSeq (Marsella et al., 2012) and low-quality reads filtered out using Trimmomatic (Bolger et al., 2014) and Sickle (Joshi and Fass, 2011). Cutadapt (Martin, 2011) was used to remove adapter sequences and rRNA transcripts were identified and removed *in silico* using sortMeRNA (Kopylova et al., 2012). Sequenced reads were identified and counted by mapping against a publicly available station ALOHA metagenomic reference gene catalog (Mende et al., 2017); https://www.imicrobe.us/#/projects/263, using the LAST algorithm (Kiełbasa et al., 2011), by nucleotide homology search with a cutoff of 50 bits and 90% identity. An additional cutoff was applied such that in order to be included in the time-series analyses, any given gene transcript was required to be present in a minimum of four transcripts per time point, averaged across all time points. The output of transcript mapping against the Station ALOHA gene catalog was a count table of transcripts mapping to genes across time for each dataset. Count tables were normalized for downstream analysis including the RAIN periodicity test and NMDS of genes based on expression levels in the following way: transcript counts in each taxonomic bin were normalized by the total number of transcripts in that taxon bin.

### Sequence and Data Deposition Accession Numbers

The RNA sequences reported in this study have been deposited in the NCBI Sequence Read Archive under project number: PRJNA545144. The transcript annotation count tables have been deposited to Figshare, with the following doi’s: doi:10.6084/m9.figshare.9250766.v1, doi:10.6084/m9.figshare.9250763.v1, doi:10.6084/m9.figshare.9250769.v1, doi:10.6084/m9.figshare.9250760.v1, doi:10.6084/m9.figshare.9253424.v2, doi:10.6084/m9.figshare.9250754.v1, doi:10.6084/m9.figshare.9250757.v1, doi:10.6084/m9.figshare.9250751.v1.

### Rhythmicity Analysis Incorporating Non-parametric Methods

In order to identify genes with statistically significant 24-h periodicity in each dataset, the longitudinal method of the RAIN test (Thaben and Westermark, 2014) was applied to within-taxon normalized transcript counts of each gene. The RAIN algorithm detects repeating patterns with a user-specified period in time-series data, designed to include both symmetric as well as unsymmetrical oscillations. RAIN is differs from other parametric tests such as harmonic regression, by avoiding the assumption of an underlying model (Thaben and Westermark, 2014). Instead of testing against simple umbrella alternatives, RAIN works by selecting the best fit against a richer set of alternatives, and can accommodate multiple rising and falling intervals of arbitrary lengths. To compensate for type I errors following RAIN analyses, a false discovery rate (FDR) correction was applied to the set of p vales from each dataset using a cutoff of 0.05.

### Ordination Methods

Two-dimensional ordination methods were used to visually compare all samples and all genes to one another based on count table data. The first two components of eigenvalue decomposition (PCA) were used to display samples relative to one another in 2D space based on transcript composition. In the PCA in the main body of the article, counts were averaged across samples that were collected at the same time of day over multiple days. A similar figure, but with each sample represented individually, can be seen in the supplement. To compare genes in each dataset to one another based on their expression levels over time, non-metric multidimensional scaling (NMDS) was performed on Euclidean distance matrices constructed from within-taxon normalized and scaled (0–1) transcript counts.

## Results

### Hydrography

**Figure 1:**
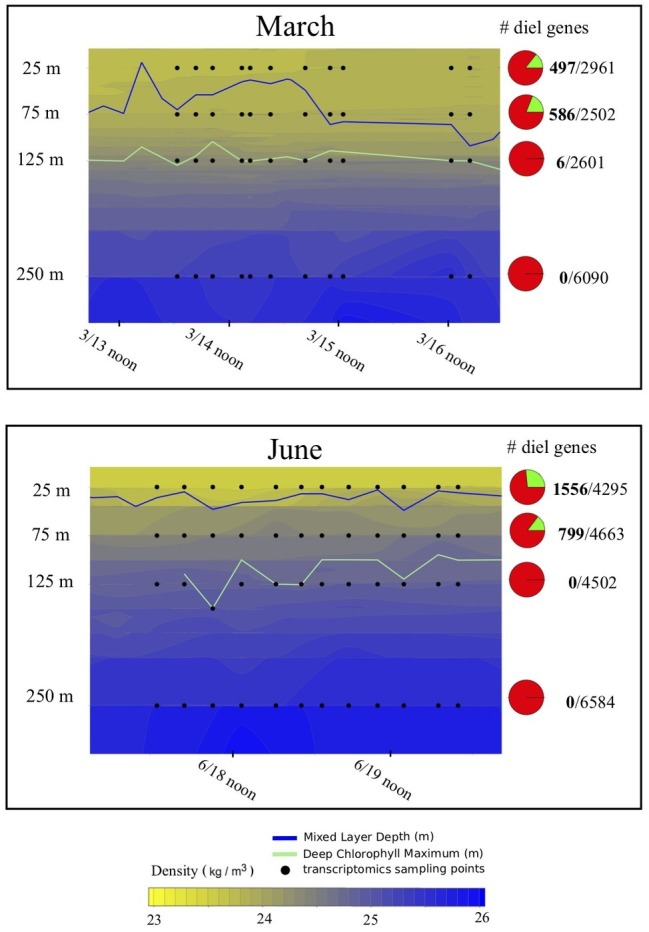
Hydrography and total proportion of transcriptomic periodicity at different depths. Depth profiles of density across the sampling periods during the two cruises included in the analysis, illustrating the shallower mixed layer during the June relative to the March cruise. Pie charts indicate the overall proportions of expressed genes exhibiting statistically significant 24-h periodicity (green wedge in the pie chart) in each dataset according to the RAIN algorithm. Numbers to the right of the pie chart indicate total number of periodic genes (in bold), over the total number of genes detected. Blue lines indicate the mixed layer depth across each sampling period, green lines indicate the depth of the deep chlorophyll maxim, and black dots indicate sampling points for transcriptomic analysis.

Upper water column hydrography during both March and June expeditions was typified by high solar energy inputs at the surface (Supplementary Figure S1A) decaying exponentially with depth (Supplementary Figure S1B). As is typical for this region (Letelier et al., 2017), the deep chlorophyll maximum (DCM) was observed at around 125 m during both cruises (Figure 1). One major difference between the two cruises was the depth of the upper mixed layer (as defined by the Levitus, 1982 as the change in potential density threshold of 0.125 kg m^−3^). During the June expedition, shallower mixing was typical of NPSG climatology, reaching to just below 25 m. In contrast, during the March expedition, the upper mixed layer extended to below 75 m, encompassing our two upper sampling depths. Nutrient concentrations were expectedly low (submicromolar levels) in the upper mixed layer during both cruises (Supplementary Figure S2), but another difference between cruises was the depth of the nutricline, defined as the depth below which nitrate+nitrite concentrations exceed 50 nM (Carmeño et al., 2008), which was significantly shallower during the June cruise (112 ± 12 m) than during the March cruise (131 ± 18 m, two-tailed t-test based on 148 observations: *p* = 0.002).

### General Patterns of Transcript Relative Abundances

**Figure 2:**
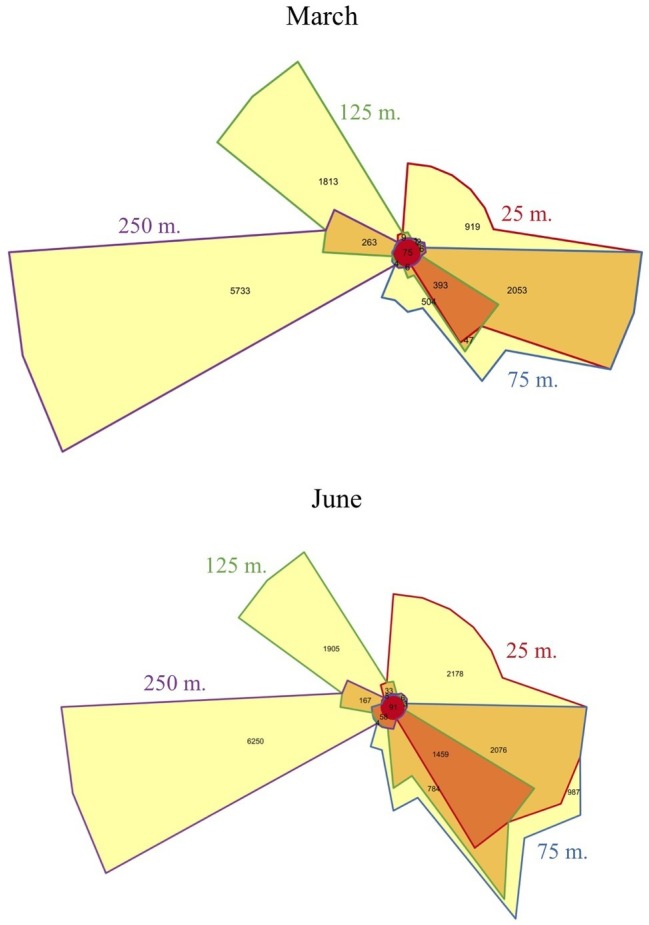
Venn diagrams of transcripts matching specific genes at each depth sampled. The diagram depicts the number of genes that mapped to transcripts in each depth. Overlap between any two depths is shown by the color of their outline (e.g., gene transcripts shared between 125 and 250 m number 263 are shown in the wedge having the half purple, half green outline). Distinct sets of gene transcripts appeared below the euphotic zone during both cruises, as well as an increase in the number of unique genes expressed in the shallower mixed layer in June at 25 m, relative to 25 m in March.

**Figure 3:**
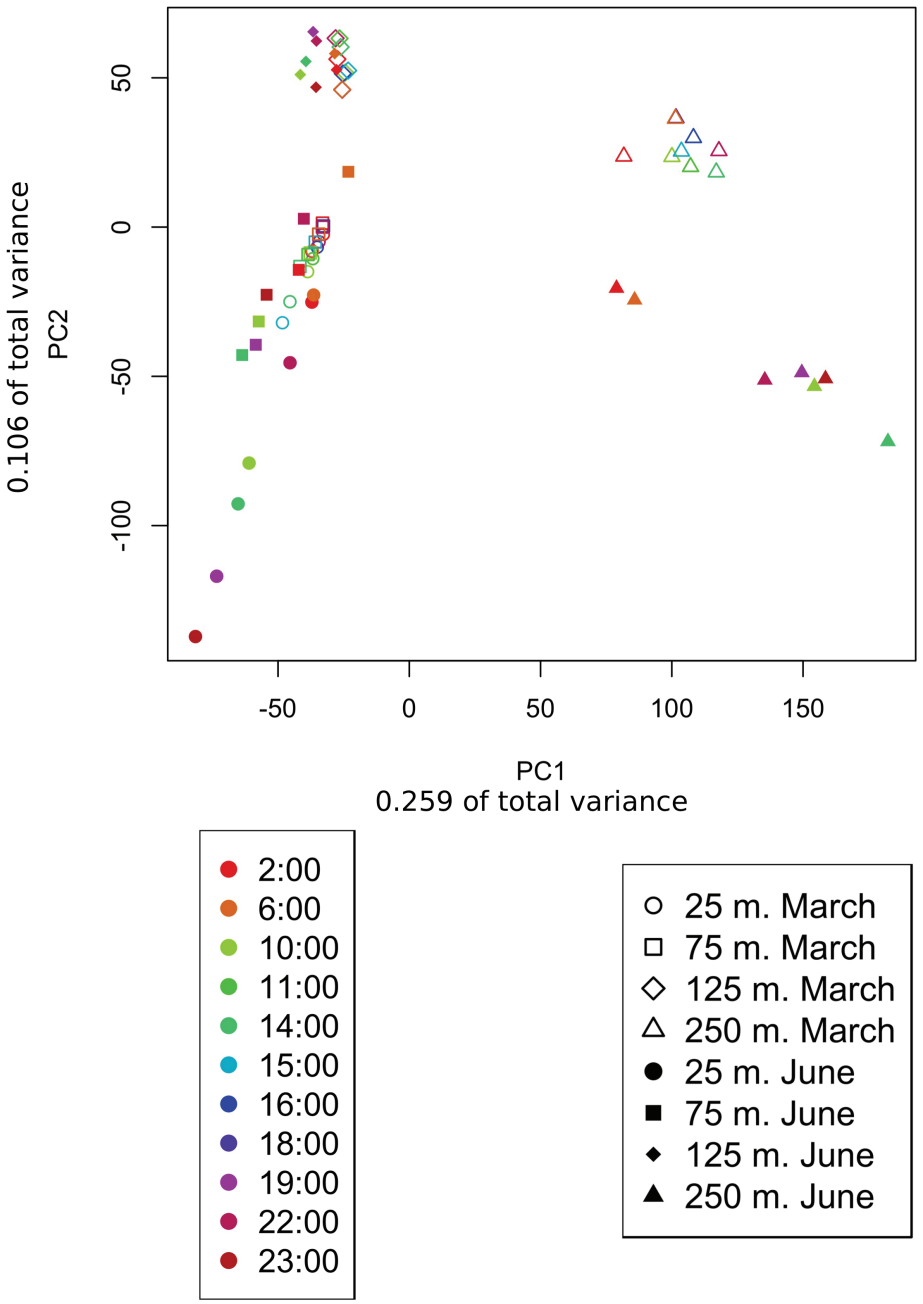
Principle component analysis of samples based on transcript counts over time. PCA arranges samples in space based on their similarity with respect to genes present and their relative expression levels. Transcript counts were averaged across samples taken at the same time over multiple days. The shape of the point indicates the sample depth, color indicates sample time, and shading indicates whether the sample belonged to the March or the June dataset. Samples clustered primarily based on depth sampled and secondarily based on the time of year (March vs. June). About 25 and 75 m samples cluster together, especially during March when both depths were encompassed in the upper mixed layer.

Metatranscriptomic sequencing of 92 samples across two diel time series at four depths (25, 75, 125, and 250 m) yielded a total of 5,193,326 quality-controlled transcripts mapping to 18,757 reference genes from the Station ALOHA gene catalog (Mende et al., 2017). About half of these gene transcripts were observed during both March and June (Supplementary Figure S3), and many of the same patterns were observed during both expeditions. An anticipated shift in transcriptome gene content and dynamics (Shi et al., 2010) was observed between the euphotic zone (25, 75, and 125 m) and the mesopelagic zone (250 m). As expected, community transcripts in the mesopelagic were distinct from those in the euphotic zone, and over 90% of the genes transcribed at 250 m were undetected in shallower transcriptomes (Figure 2). In addition, principal component analysis (PCA, Figure 3) of gene expression levels over time revealed that samples clustered primarily with respect to depth, with the two most distinct clusters consisting of 250 m samples from the two expeditions. Finally, a global shift in expression patterns between the euphotic and mesopelagic was also reflected in the significant decrease in the variance of transcript levels over time at 250 m relative to euphotic depths (*p* = 0.003, two-sample *t*-test; Supplementary Table S1).

Within the euphotic zone, gradual shifts in community structure and function appear to have been influenced by both depth and mixed layer dynamics, perhaps scaling to the time-integrated solar input received by a given parcel of water over time (greatest in the shallower 25 m June mixed layer). For example, in March, when both upper depths were encompassed in the upper mixed layer, 25 and 75 m samples shared more expressed genes (Figure 2), and clustered more closely together based on gene transcript abundances (Figure 3) than in June. In contrast, the shallower, more highly irradiated upper mixed layer in June that extended only through the 25 m depth, exhibited the largest numbers of unique gene transcripts among euphotic zone datasets (Figure 2).

In addition to facilitating comparison of samples from different depths, PCA ordination of samples based on gene expression levels (Supplementary Figures S4, S5) allowed the comparison of different samples within a given dataset. Samples from the same cruise and depths taken at the same or similar times of day across multiple days, tended clustered together, especially in euphotic zone samples (Supplementary Figures S4, S5).

### Vertical Distribution of Taxon Transcripts

**Figure 4:**
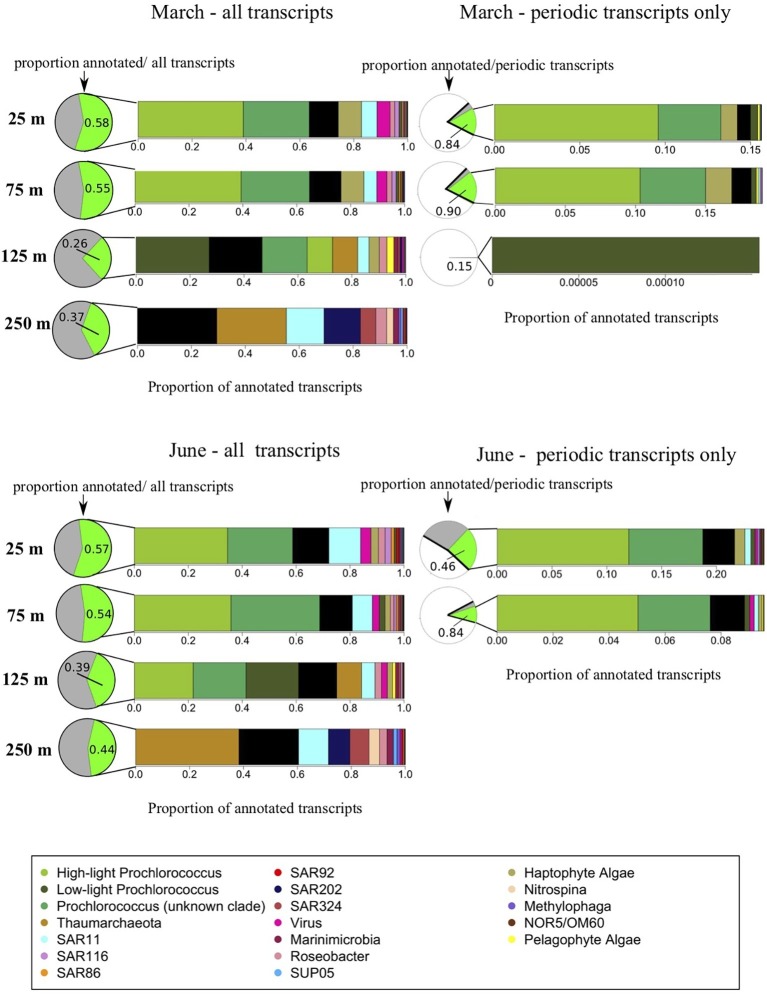
Taxonomic origins of gene transcripts. This figure illustrates taxonomic compositions of the meta-transcriptomes. Pie charts indicate the relative abundances of annotated vs. unknown transcripts while bar charts indicate the relative taxonomic composition of annotated transcripts, averaged across each time series. The March cruise is shown on the top two panels, the June cruise on the bottom two panels. The two panels on the left represent the entire metratranscriptomes, illustrating a gradual shift in transcriptomically active taxa with depth through the euphotic zone below the upper mixed layer, as well as a major change in taxonomic composition below the euphotic zone. In pie charts next to the two panels on the left, the green portion represents, at each depth, the proportion of all transcripts with known annotations, and gray portion indicates the proportion of all transcripts which are of unknown origin. The two panels on the right represent just the significantly periodic transcripts according to the RAIN algorithm, illustrating the dominance of phytoplankton within the significantly periodic pool. In the pie charts next to the panels on the right, the white portions represent non-periodic transcripts, the gray corresponds to significantly periodic transcripts with no functional or taxonomic annotation, and green are significantly periodic transcripts mapping to known annotated genes.

About half the transcripts included in the analysis could be annotated and assigned to known taxonomic and functional gene categories (Figure 4; Mende et al., 2017). The largest proportions of annotated transcripts originated from 25 m, and the largest proportion of unknown transcripts from the DCM (125 m) (Figure 4). The abundance of unannotated transcripts at 125 m is expected given that species diversity (and associated uncharacterized taxa) peak around 125–200 m at Station ALOHA (Mende et al., 2017). As is typical for gene expression data (Ueda et al., 2004; Stewart et al., 2011), distributions of expression across individual genes, and consequently across functional and taxonomic groups, roughly followed a power law with a just a few members dominating expression and a long tail consisting of many individuals with few copies. This power law distribution, combined with logistically restricted sequencing depth, limited our analyses to the 17 most transcriptionally abundant taxonomic groups across the 4 depths sampled (Figure 4; Tables 1, 2).

**Table 1:** Percentage of annotated transcripts mapping to each taxa in the March cruise.

*Taxon	25 m	75 m	125 m	250 m
High-light *Prochlorococcus*	39.01% (0.42)	39.34% (0.48)	9.45%	0
*Prochlorococcus*, unknown clade	24.43% (0.26)	25.36% (0.34)	16.68%	0
Other	10.80% (0.12)	11.72% (0.21)	19.63%	29.46%
Thaumarchaeota	0.01%	0.04%	9.33%	25.75%
SAR11	5.79% (0.015)	4.83% (0.05)	4.18%	13.97%
Low-light*Prochlorococcus*	1.02% (0.71)	1.02% (0.66)	27.11% (0.01)	0.22%
Haptophyte	8.60% (0.19)	8.44% (0.41)	3.81%	0
Virus	4.79% (0.01)	3.72% (0.01)	0.65%	0.26%
SAR202	0	0	0.72%	13.51%
*Roseobacter*	1.74%	1.88% (0.01)	2.83%	4.01%
SAR324	0.15% (0.06)	0.33%	0.75%	5.72%
SAR116	1.64% (0.02)	1.53% (0.15)	0.34%	0.10%
*Marinimicrobia*	0.11%	0.25%	1.47%	1.88%
*Nitrospina*	0	0	0	2.53%
SAR86	0.54%	0.57%	0.17%	0.35%
*Methylophaga*	0.47%	0.18%	0	0.87%
SUP05	0.19%	0.19%	0.10%	0.79%
Pelagophyte	0.28% (0.80)	0.31% (0.83)	2.62%	0
SAR92	0.35% (0.04)	0.22%	0	0.51%
NOR5/OM60	0.07%	0.07%	0.17%	0.06%

* *Values indicate percent of total annotated transcripts across all time points belonging to each of the taxonomic groups. Proportions of each taxon’s transcripts exhibiting significant diel periodicity according to the RAIN test are indicated in parentheses. The absence of a value in parentheses indicates that no transcripts belonging to that taxonomic group were periodic in that dataset*.

**Table 2:** Percentage of annotated transcripts mapping to each taxa in the June cruise.

*Taxon	25 m	75 m	125 m	250 m
High-light *Prochlorococcus*	34.59% (0.60)	35.83% (0.26)	21.81%	0.03%
*Prochlorococcus*, unknown clade	24.15% (0.49)	32.92% (0.15)	19.61%	0
Other	13.42% (0.38)	12.07% (0.19)	14.18%	22.11%
Thaumarchaeota	0.04%	0.12%	9.17%	38.32%
SAR11	11.76% (0.08)	7.46% (0.04)	5.00%	11.09%
Low-light *Prochlorococcus*	0.84% (0.81)	2.13% (0.16)	19.46%	0.13%
Haptophyte	2.74% (0.60)	1.93% (0.12)	1.91%	0
Virus	3.82% (0.10)	2.54% (0.11)	2.20%	0.58%
SAR202	0	0	0	7.92%
*Roseobacter*	2.51% (0.02)	1.38% (0.02)	2.38%	2.81%
SAR324	0.92% (0.29)	0.59% (0.01)	0.86%	7.10%
SAR116	2.26% (0.11)	0.88%	0.63%	0.12%
*Marinimicrobia*	0.42%	0.58%	1.05%	2.35%
*Nitrospina*	0	0	0	3.90%
SAR86	1.13% (0.06)	0.69% (0.02)	0.32%	0.42%
*Methylophaga*	0.40% (0.07)	0.22%	0.06%	1.14%
SUP05	0.30% (0.55)	0.19%	0.12%	1.36%
Pelagophyte	0.12% (0.80)	0.24% (0.41)	1.11%	0
SAR92	0.82% (0.17)	0.15%	0.04%	0.55%
NOR5/OM60	0.27% (0.43)	0.10%	0.11%	0.08%

* *Values indicate the percent total annotated transcripts across all time points belonging to each taxonomic group. Proportions of each taxon’s transcripts exhibiting significant diel periodicity according to the RAIN test are indicated in parentheses. The absence of a value in parentheses indicates that no transcripts belonging to that taxonomic group were periodic in that dataset*.

As expected, all upper mixed layer samples (25 and 75 m in March, and 25 m in June) shared remarkably similar distributions of taxonomic transcript annotations (Figure 4; Tables 1, 2). *Prochlorococcus* dominated upper mixed layer transcript pools at an average of 63.3 ± 3.24% of known transcript annotations between the three upper mixed layer datasets. The remaining taxonomic groups, in order of decreasing relative transcript abundance, were: haptophyte algae, *Pelagibacter* (SAR11), viruses, Alphaproteobacteria related to *Roseobacter*, *Puniceispirillum* (SAR116), and the Gammaproteobacteria SAR86 clade (Figure 4; Tables 1, 2). Less abundant taxonomic groups detected in upper mixed layer transcriptomes, that comprised less than 2% of the annotated transcripts were (in no particular order): Deltaproteobacteria clade SAR324, Chloroflexi-related SAR92, methylotrophic Gammaproteobacteria related to *Methylophaga*, pelagophyte algae, *Marinimicrobia,* the Gammaproteobacteria SUP05 clade, phototrophic, anoxygenic aerobic Gammaproteobacteria NOR5/OM60 and the Thaumarchaeota (Figure 4; Tables 1, 2).

Distributions of taxonomic annotations just below the upper mixed layer at 75 m in June were still quite similar to those in the mixed layer, with some shifts that might be expected from a decrease in solar energy input, such as an increase in low-light *Prochlorococcus* and a decrease in transcript abundances of haptophyte eukaryotic algae (Figure 4; Table 2). In congruence with previous reports, taxonomic distributions of transcripts in DCM (125 m) samples were still dominated by the same general taxa as 25 and 75 m samples, but also exhibited certain taxonomic signatures characteristic of the mesopelagic community, including increases in relative transcript abundances of Thaumarchaeota and *Marinimicrobia,* and the appearance of the SAR202 cluster (Figure 4; Tables 1, 2). Some taxonomic signatures were unique to this intermediate depth, including relative transcript maxima in pelagophyte algae and low-light *Prochlorococcus*, and minima in *Methylophaga*, SUP05, and SAR92 transcripts (Figure 3; Tables 1, 2).

Consistent with prior work (Shi et al., 2010), taxonomic annotations in mesopelagic waters (250 m) were distinct from those in euphotic waters. *Prochlorococcus* and SAR116 transcripts fell to below 1% of annotated transcripts at 250 m, and were replaced by Thaumarchaeota, which represented 32.04 ± 8.89% of transcripts at 250 m on average, between March and June (Figure 4; Tables 1, 2). SAR11 was the second-most abundant group at 250 m, comprising up 12.5 ± 2.04% of 250 m transcripts), while SAR92 and NOR5/OM60 maintained fairly even relative proportions across the 4 depths (Figure 3; Tables 1, 2). *Marinimicrobia*, SUP05, and SAR324 increased in relative transcript abundance with depth. SAR202, which was not detected in the upper mixed layer, was the third most transcriptomically abundant taxonomic group at 250 m (after Thaumarchaeota and SAR11), comprising an average of 10.7 ± 3.95% of annotated transcripts at that depth. Also absent from the upper mixed layer, the nitrite-oxidizing bacterial genus *Nitrospina* made up around 3% of the 250 m transcript pool.

### Global Comparison of Diel Transcript Oscillation With Depth

With the exception of six periodic genes detected at 125 m in March, three of which were annotated as low-light *Prochlorococccus,* and three of unknown taxonomic origin, statistically significant periodicity detected using the RAIN test was largely confined to 25 and 75 m (Figure 1; Table 1). During the March cruise, when deeper mixing resulted in both 25 and 75 m depths being contained in the upper mixed layer, samples from these two surface depths contained the most similar proportions of periodicity between any two depths (Figure 1). A slightly reduced periodic signal at 25 relative to 75 m in March may be an artifact of sampling a less stable density gradient over time at 25 m (Figure 1), and can also be implied from the relative tightness of the clustering between points from each depth on the PCA (Supplementary Figures S4, S5). During the June cruise, when shallower mixing was observed, the proportion of periodically transcribed genes in the shallow mixed layer (25 m) was more than twice the proportion of periodicity just below that mixed layer at 75 m (Figure 4). Overall, the change in proportion of overall transcript periodicity with depth tended to mirror the vertical change in daily integrated light flux – with parcels of water overturning in a shallower mixed layer receiving more light over the course of a day than those overturning in a deeper mixed layer (Figure 1).

Many of the genes that were expressed with significant diel periodicity at 25 and/or 75 m were also detected at 125 m, but not with statistically significant periodicity (20% in Mach and 44% in June), providing clear evidence for diminished diel periodicity with depth. Less than 1% of genes found to be transcribed with diel periodicity in euphotic waters were detected at all in 250 m transcriptomes. It is possible that some transcript diel oscillations occurred at these depths, but they were below the limits of detection. Our observations suggest that below the euphotic zone, microbial communities are much less temporally synchronized around diel cycles, compared to those in euphotic zone.

### Periodic Gene Expression Across Depth and Season in Phytoplankton

Transcripts most similar to homologues from high-light clades of *Prochlorococcus* (referred to herein as “high-light *Prochlorococcus*”) were responsible for the vast majority of the statistically significant transcript periodicity overall (Figure 4). Consequently, the change in relative proportions of high-light periodicity vertically through the water column paralleled that previously described for whole community, roughly scaling in magnitude to the 24-h integrated light signal (Figure 4; Tables 1, 2). Where detected, high-light *Prochlorococcus* periodicity ranged from a minimum of 26% of high-light transcripts at 75 m below the mixed layer in June, to a maximum of 60% of high-light transcripts in the shallow mixed layer in June (Table 2). In March, high-light *Prochlorococcus* periodicity in the upper mixed layer averaged just under 50% of high-light transcripts between the 25 and 75 m depths (Table 1). These values are consistent with previous reports in surface waters (Ottesen et al., 2014; Aylward et al., 2015). Even though transcripts from high-light *Prochlorococcus* clades were present at the DCM (125 m), albeit in lower relative abundances than at shallower depths, none were detected as statistically periodic at that depth by the RAIN test (Figure 4; Tables 1, 2).

For low-light clades of *Prochlorococcus*, the strength of the periodic signal generally appeared to be even more sensitive to changes in time-integrated light input than that of high-light strains. Although, at 25 and 75 m, overall proportions of transcripts mapping to low-light relative to high-light clades of *Prochlorococcus* were much smaller, a relatively larger proportion of those low-light *Prochlorococcus* transcripts was significantly periodic (Figure 4; Tables 1, 2). Over 80% of low-light *Prochlorococcus* transcripts in the shallow mixed layer in March (Table 1) exhibited diel periodicity, while approximately 70% were periodic across the deeper mixed layer in June (Table 2). Below the upper mixed layer, low-light *Prochlorococcus* periodicity diminished to around 2% of all low-light transcripts at 75 m in June (Table 2). At the DCM (125 m), low-light *Prochlorococcus* was the only known taxonomic group to exhibit significantly periodic expression, and only during the March cruise (Figure 4; Table 1). The periodic *Prochlorococcus* genes at 125 m in March were from the photosynthesis functional category (Supplementary Figure S6).

*Prochlorococcus* transcript periodicity associated with metabolic functions (Supplementary Figures S6, S7).

**Figure 5:**
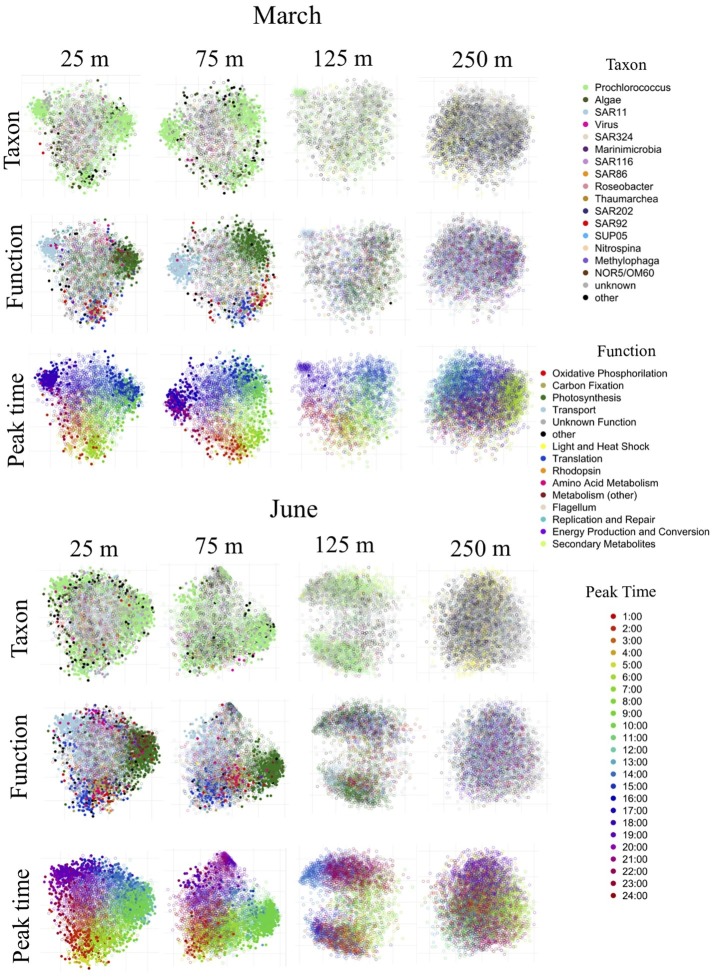
NMDS clustering of genes based on distance matrix of transcript abundance patterns over time. This figure illustrates two-dimensional non-metric multidimensional scaling of all genes in each dataset based on pairwise distances between them. Each point represents a gene in the dataset and the distances between points represent their similarity in expression profiles over time. Each NMDS plot is shown three times, colored differently each time: by taxonomic annotation, by functional annotation and by peak time. Filled-in circles indicate that the temporal expression of that gene was periodic according to the RAIN algorithm. Hollow circles were not significantly diel. Significantly periodic genes near the surface belonged mostly to *Prochlorococcus* and formed distinct clusters, differentiable by their functional annotations and peak transcript abundance times. Diel patterns of transcript abundance diminished with depth, and were undetectable below the euphotic zone.

Two-dimensional NMDS ordination of genes in each dataset based on taxon-normalized transcript counts over time revealed that the majority of genes expressed with significant periodicity, most of which belonged to *Prochlorococcus,* fell into one of three clusters which were differentiable by functional annotation (Figure 5). The clusters were most prominent in the upper mixed layer of the March cruise (25 and 75 m). The most diverse diel *Prochlorococcus* cluster, peaking around sunrise, was dominated by genes associated with the following functional groups: translation, carbon fixation, oxidative phosphorylation, and amino acid metabolism (Figure 5; Supplementary Figures S6, S7). The vast majority of periodic transcripts in the translation category encoded a variety of ribosomal proteins, with a few genes also encoding elongation factor proteins. Within the carbon fixation functional category, the most common periodic transcripts mapped to genes encoding ribulose 1,5-biphsophate carboxylase/dehydrogenase (rubisco), fructose bisphosphate aldolase, and phosphoribulokinase and carboxysome proteins. In the oxidative phosphorylation category, the vast majority of periodic transcripts mapped to ATP synthase. Although concentrated around sunrise, some *Prochlorococcus* periodic transcripts associated with oxidative phosphorylation and amino acid metabolism also peaked throughout the day, and some associated with translation peaked throughout the night (Figure 5; Supplementary Figures S6, S7). *Prochlorococcus* high light/high heat inducible “shock” genes, the most common being superoxide dismutase, also tended to peak in expression before sunrise or during the first part of the day (Figure 5; Supplementary Figures S6, S7). A second cluster of periodic *Prochlorococcus* genes (Figure 5) was dominated by photosynthesis genes peaking either just before noon or in the afternoon (Supplementary Figures S6, S7). The most transcribed periodic gene in the photosynthesis category encoded the photosystem II 44 kda reaction center protein. Photosystem II D1 and D2 proteins and photosystem I P700 chlorophyll A apoprotein A2 were also common periodic transcripts in the photosynthesis category. Less common but also included among periodic genes in the photosynthesis category were plastocyanin and cytochrome b559 subunit proteins. The third periodic *Prochlorococcus* cluster (Figure 5) was composed mostly of transporter genes peaking in expression at sunset. The vast majority of these periodic transporters mapped to genes for ammonium transporters. Although the 25 and 75 m samples were both encompassed in the deeper March mixed layer, peak times of many of these periodic transporter transcripts were shifted to slightly earlier in the day at 75 m relative to 25 m (Supplementary Figures S6, S7), indicating a possible regulatory mechanism that modulates timing of expression of transporters based on instantaneous light input.

The highly-irradiated shallow mixed layer at 25 m in June exhibited an overall increase in *Prochlorococcus* diel periodicity relative to the March upper mixed layer, with periodicity extending across a greater variety of genes. This included increases in periodicity of light and heat shock transcripts, various metabolic transcripts, replication and repair transcripts (Figures 4, 5; Supplementary Figures S6, S7). A larger variety of genes in the translation category were periodic at 25 m in June, most notably co-chaperone GroES, as well as various reductases and cell division protein FtsH. In the photosynthesis category, several different photosystem II manganese-stabilizing polypeptides were found to be periodic only in the June 25 m dataset. Another difference between March and June was a shift in the peak times of certain periodic *Prochlorococcus* genes. Most notably, the ammonium transporters appeared to closely track the timing of the sunset, which was later in the day in June relative to March. In addition, afternoon peak times of some *Prochlorococcus* photosynthesis genes shifted slightly to earlier in the day in June relative to March (Supplementary Figures S6, S7).

Below the upper mixed layer, a decrease and eventual disappearance of the three major functional clusters of periodic *Prochlorococcus* genes was observed (Figure 5). Just below the mixed layer at 75 m in June, when both mixing and the nutricline were shallower than in March (Figure 1; Supplementary Figure S3), the appearance of clusters was reduced, accompanying an overall decrease in periodicity, with the greatest loss of periodicity observed in the evening-peaking ammonium transporter group (Figure 5; Supplementary Figures S6, S7). Furthermore, some unique periodic signals of the 75 m June dataset were associated with nitrogen metabolism. The 75 m June dataset also exhibited a larger variety of periodic genes from the amino acid metabolism category including: phosphoglycerate dehydrogenase, gamma-glutamyl-phosphate reductase and the acetolacetate synthase catalytic subunit.

At 125 m, diel clustering was further reduced in both cruises, albeit *Prochlorococcus* transcript abundances still appeared to weakly oscillate, with photosynthesis transcripts peaking at midday, rubisco transcripts in the morning, and transporter transcripts at night (Supplementary Figures S6, S7; Table 2). However, statistically significant periodic expression in *Prochlorococcus* was detected at 125 m only in March, in several photosystem II chlorophyll-binding genes. Although ammonium transporter periodicity was the first to diminish with depth in June, during the March cruise when the nutricline was deeper and nitrite+nitrate concentrations remained at submicromolar levels past 125 m, the evening-peaking cluster of ammonium transporter transcripts was the last remaining element of 24-h organization to persist with depth (Figure 5).

Eukaryotic phytoplankton exhibited the second largest number of periodic transcripts after *Prochlorococcus* and followed many of the same general patterns of changes in periodicity with depth to *Prochlorococcus,* with one major exception being the absence of periodic evening-peaking transporters in the eukaryotes (Supplementary Figures S6, S7). In the two shallower depths where haptophyte transcript periodicity was observed, total proportions of periodicity also appeared to scale with time-integrated light inputs: highest at 25 m in the shallow June mixed layer at 60% of their total transcripts and lowest at 75 m, below that mixed layer, at 12% periodicity (Figure 4; Supplementary Figures S6, S7; Table 2). Although much less abundant than haptophytes in transcript representation, most of the pelagophyte transcripts detected (~80%) exhibited significant periodicity consistently throughout the three surface mixed layer depths of both cruises (25 and 75 m in March and 25 m in June) (Figure 4; Supplementary Figures S6, S7; Tables 1, 2). Despite the presence of eukaryotic algae at 125 m, no statistically significant diel periodicity was detected in algal transcripts at that depth, and no algal transcripts were detected at 250 m (Figure 4; Supplementary Figures S6, S7). As with *Prochlorococcus,* eukaryotic algal periodic photosynthesis transcripts tended to peak during the day, and amino acid metabolism, carbon fixation and oxidative phosphorylation periodic transcripts tended to peak around sunrise (Supplementary Figures S6, S7). Among eukaryotic algal transcripts, the functional group retaining the highest number of periodic transcripts past the upper mixed layer (at 75 m in June) was associated with carbon fixation (Supplementary Figure S7).

### Diel Transcriptional Oscillations in Heterotrophic and Chemolithotrophic Bacteria and Archaea

Consistent with previous reports, periodicity was detected across a variety of heterotrophic microbial groups, although in much smaller relative proportions than was observed for phytoplankton. Detectable diel periodicity in heterotrophic bacteria was confined to 25 and 75 m depths (Tables 1, 2; Figures 4, 5; Supplementary Figures S8, S9). The majority (9 out of 13) of microbial taxa included in our analysis exhibited some statistically significant periodicity in some dataset, in some portion of their genome. The four taxa that failed to exhibit any detectable diel periodicity – SAR202, *Nitrospina,* Thaumarchaeota, and *Marinimicrobia,* all exhibited transcript maxima below the euphotic zone (Tables 1, 2), indicating that all taxa observed to specialize in living in euphotic waters seem capable of diel synchronization. Between datasets, periodic gene expression in these heterotrophic groups had a variety of functional annotations, peaking at a variety of times throughout the day (Supplementary Figures S8, S9).

As observed in phytoplankton, the largest number and variety of heterotrophic and chemolithotrophic bacterial genes were periodic in the highly irradiated shallow mixed layer in June (25 m) (Supplementary Figure S8; Table 2). Of the nine taxa that did exhibit periodicity, four exhibited periodicity only in June (SAR86, *Methylophaga*, SUP05, and NOR5/OM60) (Supplementary Figures S8, S9; Tables 1, 2). Periodic transcripts belonging to these taxa encoded a variety of functions including, rhodopsins in SAR86 and SUP05, transport in SAR86, SUP05 and NOR5/OM60, amino acid and other metabolism in SAR86 (peaking in the afternoon), and translation and energy production and conversion transcripts in SAR92 and NOR5/OM60 (Supplementary Figures S8, S9). These observations represent one of the first reports of diel cycling in *Methylophaga*, SUP05 and NOR5/OM60 clades, and add to the growing list of diel-cycling heterotrophic or chemolithotrophic bacterial clades.

SAR11 periodicity was detected through both upper depths in both cruises. In June, SAR11 periodicity decreased with depth from 8% of SAR11 transcripts in the upper mixed layer (25 m) to 4% at 75 m (Table 2). In March, the proportion of detected SAR11 periodicity actually increased with depth from 1% at 25 m to 5% at 75 m (Table 1), perhaps due to a previously described artifact of greater mixed layer instability at 25 m relative to 75 m in March. These proportions of SAR11 transcript periodicity were similar to previously reported numbers of ~7% of SAR11 diel transcripts periodicity at 23 m (Ottesen et al., 2014). Periodic SAR11 transcripts belonged to a variety of functional groups including translation transcripts tending to peak in the morning, replication and repair transcripts tending to peak in the evening, and transport, amino acid metabolism, oxidative phosphorylation and rhodopsin transcripts peaking at various times (Supplementary Figures S8, S9). The highly irradiated 25 m June dataset saw an increased number and variety of periodic SAR11 genes, including a notable increase in periodic rhodopsin transcripts, which peaked during the first half of the day (Supplementary Figure S9).

SAR116 periodicity was observed across upper mixed layer samples. In March, at 25 m, 2.4% of SAR116 transcripts were significantly periodic, peaked in the evening, and mapped to translation and transport genes (Table 1; Supplementary Figure S8). At 75 m in March, periodic transcripts made up almost 15% of SAR116 transcripts, peaked in the morning, and were associated with transporters and rhodopsins (Table 1; Supplementary Figure S8). At 25 m in June, the 15% of SAR116 transcripts which were periodic were distributed across a variety of functional categories, including: transport, rhodopsins (peaking around sunrise), amino acid metabolism, translation, and energy production and conversion (Table 2; Supplementary Figure S9). These results confirm and extend previous studies which reported periodicity in SAR116 transcripts related to respiration, nitrogen metabolism and translation, peaking during daytime (Ottesen et al., 2014; Aylward et al., 2015).

SAR324 periodicity was observed only at 25 m in both cruises, increasing from 6.5% in March to almost 30% in June. SAR324 periodicity featured rhodopsin transcripts peaking during the first part of the day and translation transcripts peaking in the second half of the day (Tables 1, 2; Supplementary Figures S8, S9). SAR92 periodicity was also observed only at 25 m in both cruises, also increasing between March and June from 5% to almost 20% (Tables 1, 2; Supplementary Figures S8, S9). In March, the only periodic SAR92 transcripts were annotated as peroxidase precursors and peaked during the night. In June, periodic SAR92 genes peaked in expression throughout the day, and were annotated to a variety of functional groups including translation, energy production, and transport (Supplementary Figure S9). *Roseobacter* exhibited the smallest proportion of transcript diel periodicity among those taxa exhibiting periodicity, reaching a maximum of only 2.5% at 25 m in June (Tables 1, 2; Supplementary Figures S8, S9). Periodic *Roseobacter* transcripts mapped to genes as in a variety of functional categories including light harvesting, transport, and transcription (Supplementary Figures S8, S9).

The dramatic shift in community composition between the euphotic zone and the mesopelagic zone (Figure 4) was accompanied by a transition to transcripts no longer exhibiting diel patterns (Figures 1, 4; Tables 1, 2), reflecting instead a change in community structure, with increases in transcripts associated with remineralization, transport, motility, and chemolithoautotrophic nitrification (Figure 5; Supplementary Figures S8, S9). Even though a few of the taxa with transcript maxima in the mesopelagic demonstrated some capacity of diel synchronization where they were also found in the euphotic (SAR324, SUP05, and *Methylophaga)*, no statistically significant diel periodicity in transcript abundances was detected at 250 m for any taxa, and no 24-h organization was observed the ordination plots (Figure 5) or peak transcript abundance times (Supplementary Figures S6–S9).

## Discussion

This study confirms previous reports of widespread diel periodicity in transcript abundance across the most abundant and active members of the surface pelagic microbial, including *Prochlorococcus,* eukaryotic algae, *Roseobacter*, and various SAR clades, and expands the list of taxa exhibiting diel periodicity to include three less abundant members of these communities: SUP05, *Methylophaga* and NOR5/OM60. With few exceptions, most microbial taxa detected in euphotic zone, including all taxa having their transcript maxima there, demonstrated statistically significant levels of diel oscillation, underscoring the significance of the diel cycling for microbial communities in the sunlit open ocean.

Functional annotations of periodic transcripts confirmed the diversity of diel functions across this community especially in phytoplankton. As expected, clock-driven periodicity synchronized large portions of phytoplankton genomes to the 24-h cycle: transcripts associated with functions preparing cells for daytime activities, such as ribosomal proteins and carbon fixation genes, tended to peak around sunrise, photosynthesis gene transcripts tended to peak midday, and for *Prochlorococcus* but not eukaryotic algae, ammonium transporter transcripts tended to peak at sunset. Among heterotrophic and putative chemolithotrophic microbes, diel periodicity was less pronounced, and the functions and peak times of periodic genes varied between taxa and datasets. A few general trends in heterotroph periodicity were observed across datasets, and included the following: rhodopsin transcripts tended to peak before sunrise or during the first half of the day, replication and translation transcripts tended to peak in the afternoon (offset in time from the morning peak times of translation in phytoplankton), and amino acid metabolism and oxidative phosphorylation transcripts peaked over a variety of different times (Supplementary Figures S6, S7).

Diel patterns in transcript abundance were observed throughout the euphotic zone, but decreased with diminishing light availability, and were absent from the mesopelagic. The near-complete disappearance of 24-h organization at 250 m was concomitant with a shift toward a community dominated mostly by mostly mesopelagic taxa. The apparent absence of transcript diel oscillations at 250 m, even in those taxa that exhibited periodicity in shallower waters, suggests that even if 24-h patterns in other variables besides light do exist below the euphotic zone, synchronization to such patterns may not provide a significant selective advantage.

Within the euphotic zone, time-integrated light input, rather than some secondary processes occurring with 24-h periodicity, appeared to drive the strength of the periodic signal. This was suggested by the correspondence of light availability with the proportions of periodic genes, the ranges of periodic taxa and functions, and the magnitudes of diel oscillations in transcript abundance. The strongest diel signals were found in the shallower upper mixed layer at 25 m in June, followed somewhat diminished diel patterns spanning across 25 and 75 m in the deeper upper mixed layer in March. Below the upper mixed layer, periodic signals decreased dramatically at 75 m in June, and were barely detectable at 125 m in both cruises. Although most of the periodic signal 125 m was too weak and/or irregular for detection by the RAIN periodicity analysis, some 24-h organization was still apparent, though greatly reduced relative to shallower depths in the NMDS analysis (Figure 5), and plots of peak times of expression (Supplementary Figures S6, S7).

These changes in the strength of the periodic signal through the euphotic zone were mostly the result of changes in behavior of the dominant taxa, rather than community shifts. Certain taxa, like *Prochlorococcus*, exhibited a diminishing periodic signal with diminishing light levels, whereas others exhibited periodicity exclusively in highly irradiated waters despite being present throughout the euphotic zone. The shallow, highly-irradiated upper mixed layer in June particularity stood out for demonstrating a unique subset of periodic taxa and large fraction of unknown (unannotated) periodic genes. It is possible that in euphotic microbial communities, different taxa and/or genes have different thresholds of daily light input required for activation of periodicity, although the diversity of peak times, even among the heterotrophs, indicates that regulation of these genes is more complex than simply being a response to instantaneous light levels.

The occurrence of periodicity appeared to be driven primarily by light, but modulated in some cases by nutrient concentrations, further emphasizing the hypothesized relationship between periodicity and nutrient exchange/community metabolism (Aylward et al., 2015). In *Prochlorococcus,* the shallower nutricline in June corresponded to a decrease in the periodic expression of ammonium transporters at the base of the euphotic zone. The timing of the periodicity of ammonium transporter expression in *Prochlorococcus* may represent a carefully regulated balance. Although the occurrence of ammonium transporter periodicity appeared be controlled by a combination of daily-light flux and surrounding nutrient concentrations, the timing of peak expression of these genes appeared closely tied to instantaneous light levels across seasons and depths. Peak times of ammonium transporter transcript abundance varied at different times of year (appearing to closely follow seasonal shifts in the time of sunset), and throughout the water column (tending to peak slightly earlier with depth). This may reflect common regulatory mechanisms among photoautotrophs that sets the precise timing of transcript oscillations *via* instantaneous light inputs. It is important to note here that transcript abundances do not necessarily equate to the timing of specific activities or rate processes (Waldbauer et al., 2012; Rocca et al., 2015). In select cases however, transcript maxima do correspond well with downstream translational or enzymatic activities, and in those cases, they can serve as useful temporal biomarkers or environmental sensors (Wilson et al., 2017; Frischkorn et al., 2019).

In summary, our study indicates that diel patterns in transcript oscillations are absent below the euphotic zone and that within the euphotic zone, diel transcript oscillations decrease in abundance, strength and regularity with increasing depth and decreasing light levels. These variations in transcript levels in space and time suggest that diel patterns in microbial processes exist throughout the euphotic zone in both photoautotrophs and heterotrophs, where they diminish with increasing depth below the upper mixed layer. Diel transcript periodicity was most pronounced in genes whose functions relate directly to light energy acquisition, but they were also found across a variety of metabolic gene functions, the spatiotemporal distributions of which may be influenced in part by other environmental factors such as nutrient concentrations. These observations and analyses shed new light on vertical heterogeneity in daily microbial activities in the NPSG. Further resolution of the precise nature of temporally-phased microbial metabolic activities and processes and their environmental variability relative to light and nutrient levels, will benefit from direct experimental approaches in the lab and the field, in addition to greater spatiotemporal sampling resolution coupled with metabolomic, proteomic, and biogeochemical analyses.

## Data Availability Statement

The datasets generated for this study can be found in the NCBI Sequence Read Archive under project number: PRJNA545144. The RNA sequences reported in this study have been deposited in the NCBI Sequence Read Archive under project number: PRJNA545144. The transcript annotation count tables have been deposited to Figshare, with the following DOIs: doi:10.6084/m9.figshare.9250766.v1, doi:10.6084/m9.figshare.9250763.v1, doi:10.6084/m9.figshare.9250769.v1, doi:10.6084/m9.figshare.9250760.v1, doi:10.6084/m9.figshare.9253424.v2, doi:10.6084/m9.figshare.9250754.v1, doi:10.6084/m9.figshare.9250757.v1, doi:10.6084/m9.figshare.9250751.v1.

## Author Contributions

ED conceived the project and directed the study. OS collected samples in the field. Laboratory work was performed by AR and AV. Bioinformatic analysis of sequence data was conducted by AV and JE. Further data analysis and integration was done by AV, with guidance from ED. The manuscript was written by AV and ED, with critical input from OS and AR.

### Conflict of Interest

The authors declare that the research was conducted in the absence of any commercial or financial relationships that could be construed as a potential conflict of interest.

## Footnotes

^1^http://hahana.soest.hawaii.edu/hoeboe/documents/documents.html
