# Supplementary material for: Diel Oscillation of Microbial Gene Transcripts Declines With Depth in Oligotrophic Ocean Waters
Source: Front Microbiol. 2019 Sep 24;10:2191. doi: 10.3389/fmicb.2019.02191 (PMC6769238; doi:10.3389/fmicb.2019.02191)
Supplement: Supplementary file 1 [file Data_Sheet_1.PDF]

Supplementary Material

Supplementary Figures

Supplementary Figure S1A. Surface Light level measured throughout cruises represented on a 24-hour scale. Surface PAR measurements during the two cruises over the course of 24 hours (here measurements taken over the course of the sampling period are collapsed into 24 hours).

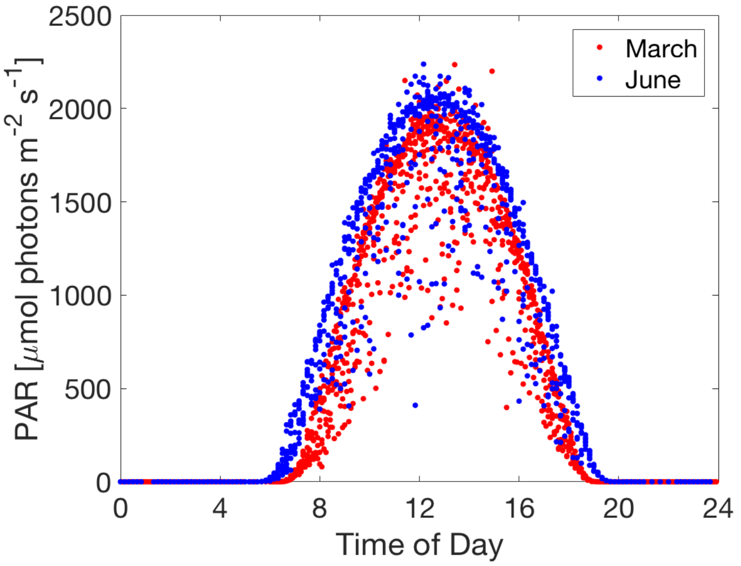

Supplementary Figure S1B. Attenuation of surface PAR as a function of depth.

Light attenuation was measured via hyperpro at various depths as describe in Methods.

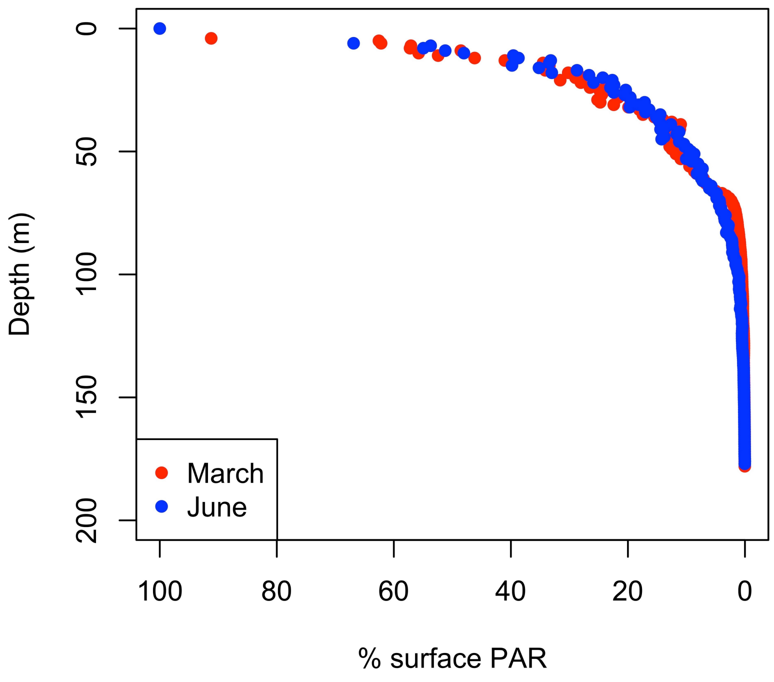

Supplementary Figure S2. Nitrate + nitrite levels versus depth.

Measurements taken over the course of the sampling period collapsed into a single depth profile. Red color represents the March cruise and blue, the June cruise.

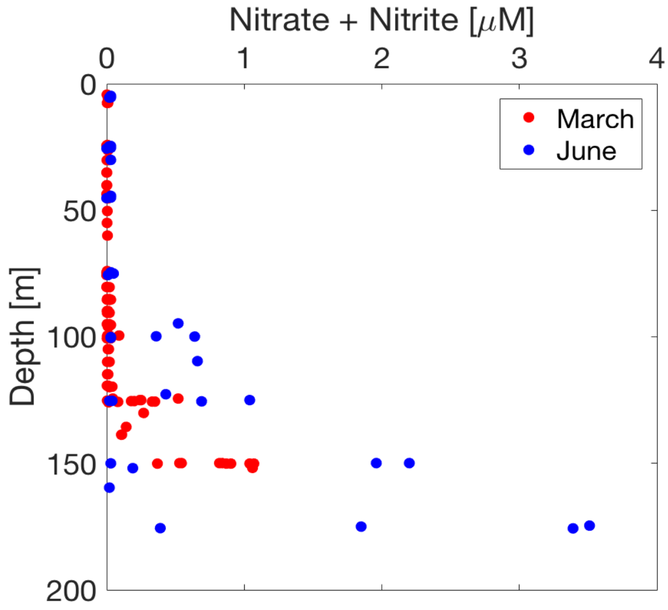

Supplementary Figure S3. Gene sets mapping to transcripts that overlap between March and June cruises.

The Venn diagram representing the overlap, between the two cruises, of all genes expressed across the depth series, where area of overlap is proportional to number of overlapping genes between the two cruises.

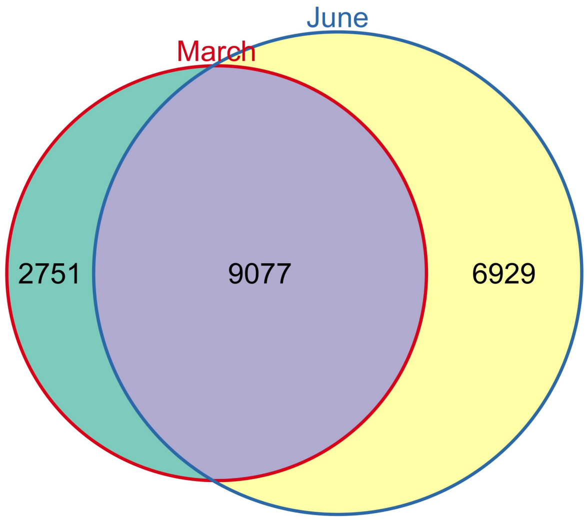

Supplementary Figure S4: PCA plots of transcript abundance distance matrices from all time points from the March cruise

Each time point from the March cruise is represented by a single point. The shape of the point (circle, square, diamond or triangle) indicates the depth the sample was collected at. The color of a point indicates time of sample collection. For each dataset, the first occurrence of a given sampling time is indicated by a hollow point, whereas the second occurrence of sampling at that time is indicated by a filled point. Here, for samples collected at 125 m and 250 m, clustering can be observed between samples taken at the same depth. 25 m and 75 m samples, on the other hand, cluster together which is consistent with the deeper upper mixed layer observed in March. Furthermore, within the upper mixed layer cluster (25 m and 75 m), 25 m samples are spread wider than 75 m samples, possibly reflecting a greater density gradient instability which may have affected our ability to detect periodicity at 25 m relative to 75 m, to some extent.

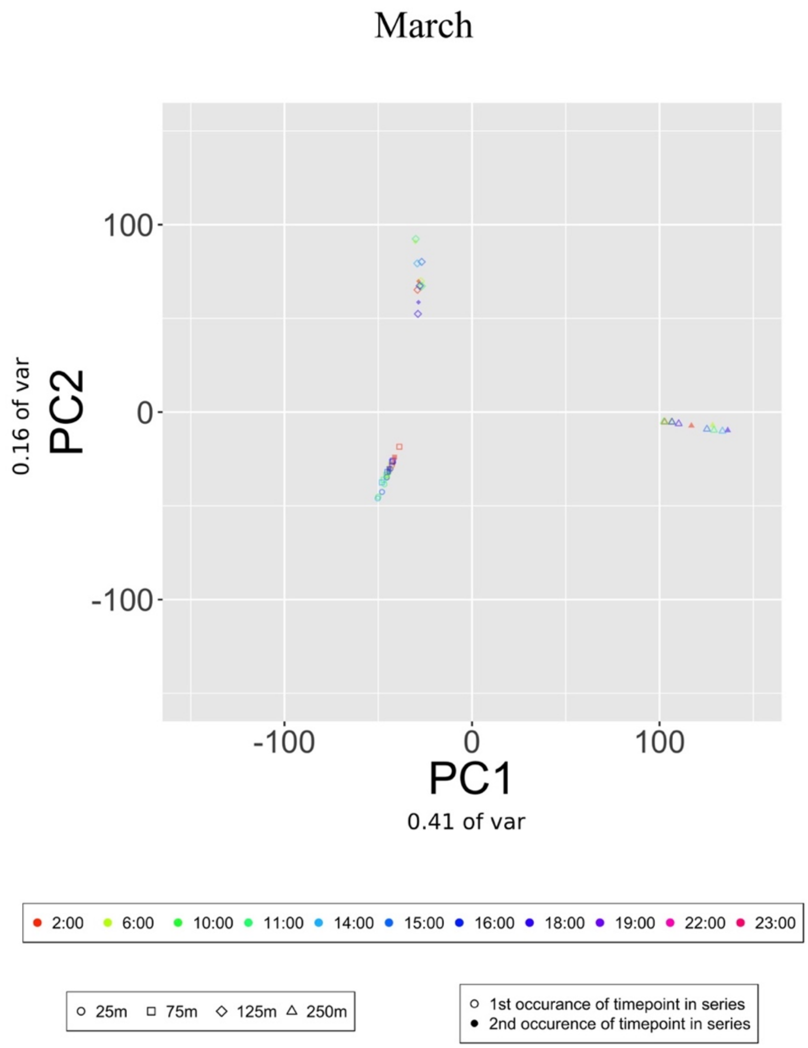

Supplementary Figure 5: PCA plots of transcript abundance distance matrices from all time points from the June cruise

Each time point from the June cruise is represented by a point in space. The shape of the point (circle, square, diamond or triangle) indicates the depth the sample was collected at. The color of a point indicates time of sample collection. For each dataset, the first occurrence of a given sampling time is indicated by a hollow point, whereas the second occurrence of sampling at that time is indicated by a filled point. In June, samples from each of the four depths cluster mostly with one another, although the larger spread at 25 m likely due to mixed layer instability near the surface.

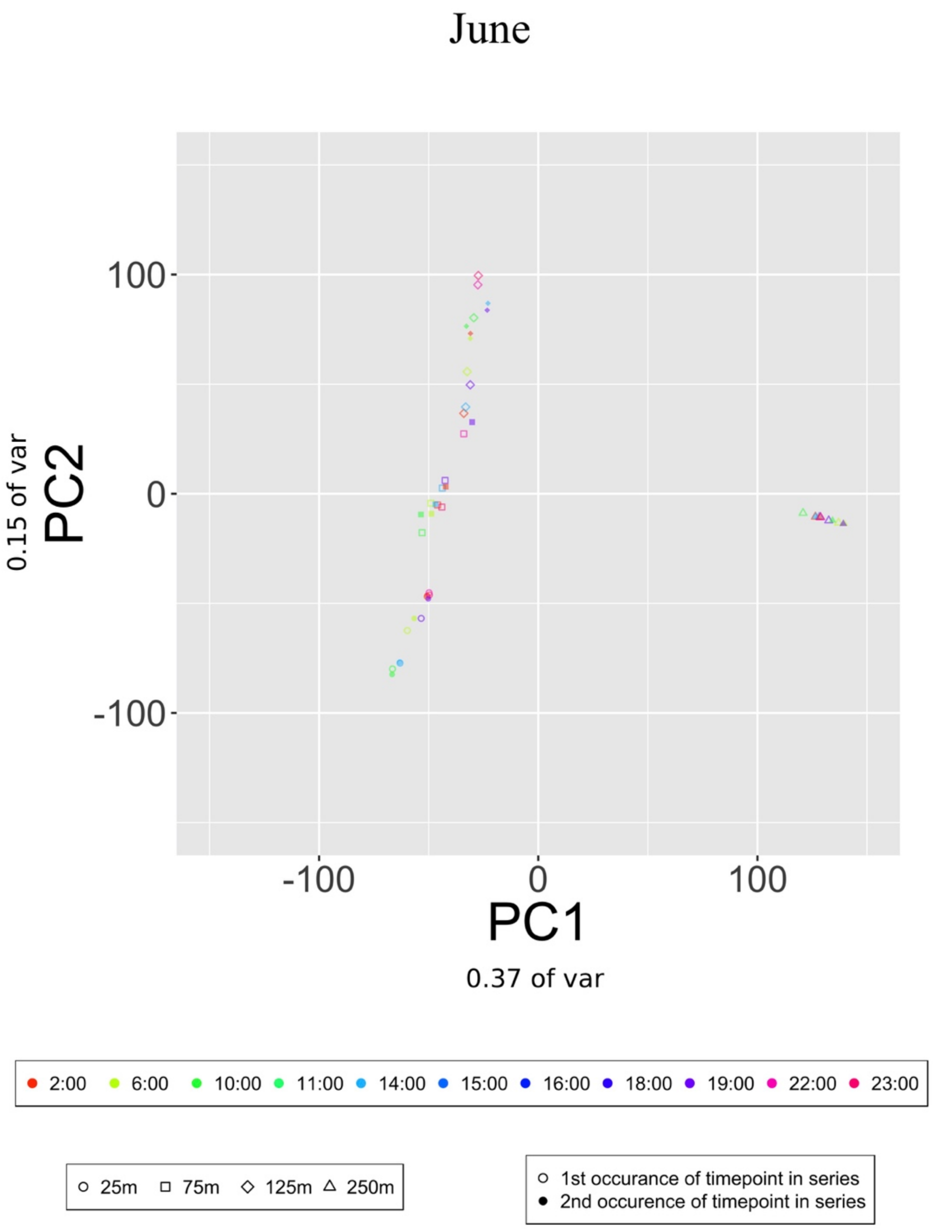

Supplementary Figure S6. Functions and peak times of major phytoplankton transcripts detected in the March cruise.

Points represent peak times of gene transcript abundance by taxa (rows) and function (color) of major phytoplankton groups during the March cruise through depth. Filled circles represent peak times of genes expressed with significant periodicity, whereas plus signs represent peak times of genes that are not significantly periodic, according to the RAIN test.

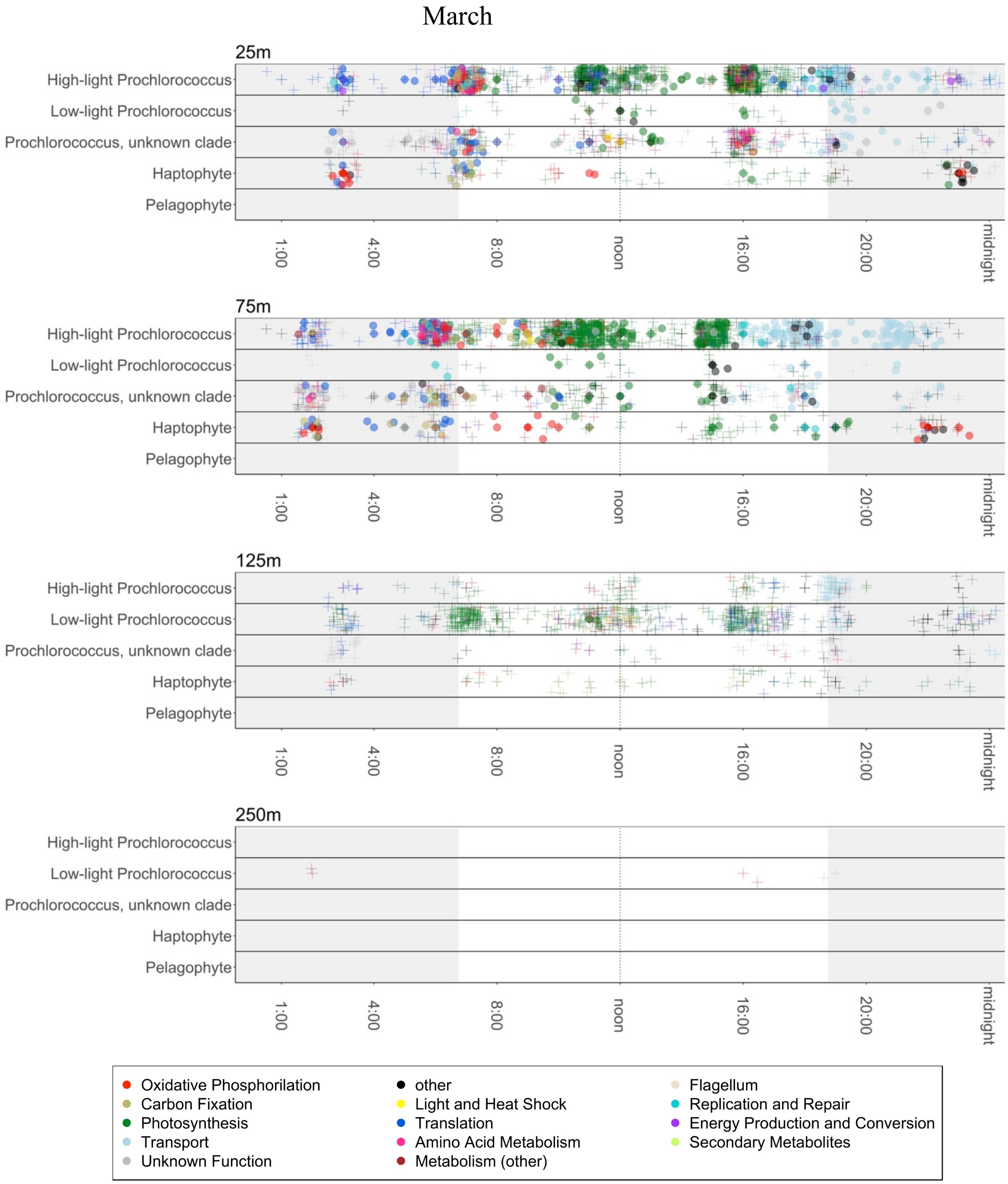

Supplementary Figure S7. Functions and peak times of major phytoplankton transcripts detected in the June cruise.

Points represent peak times of gene transcript abundance by taxa (rows) and function (color) of major phytoplankton groups during the June cruise through depth. Filled in circles represent peak times of genes expressed with significant periodicity, whereas plus signs represent peak times of genes that are not significantly periodic, according to the RAIN test.

June

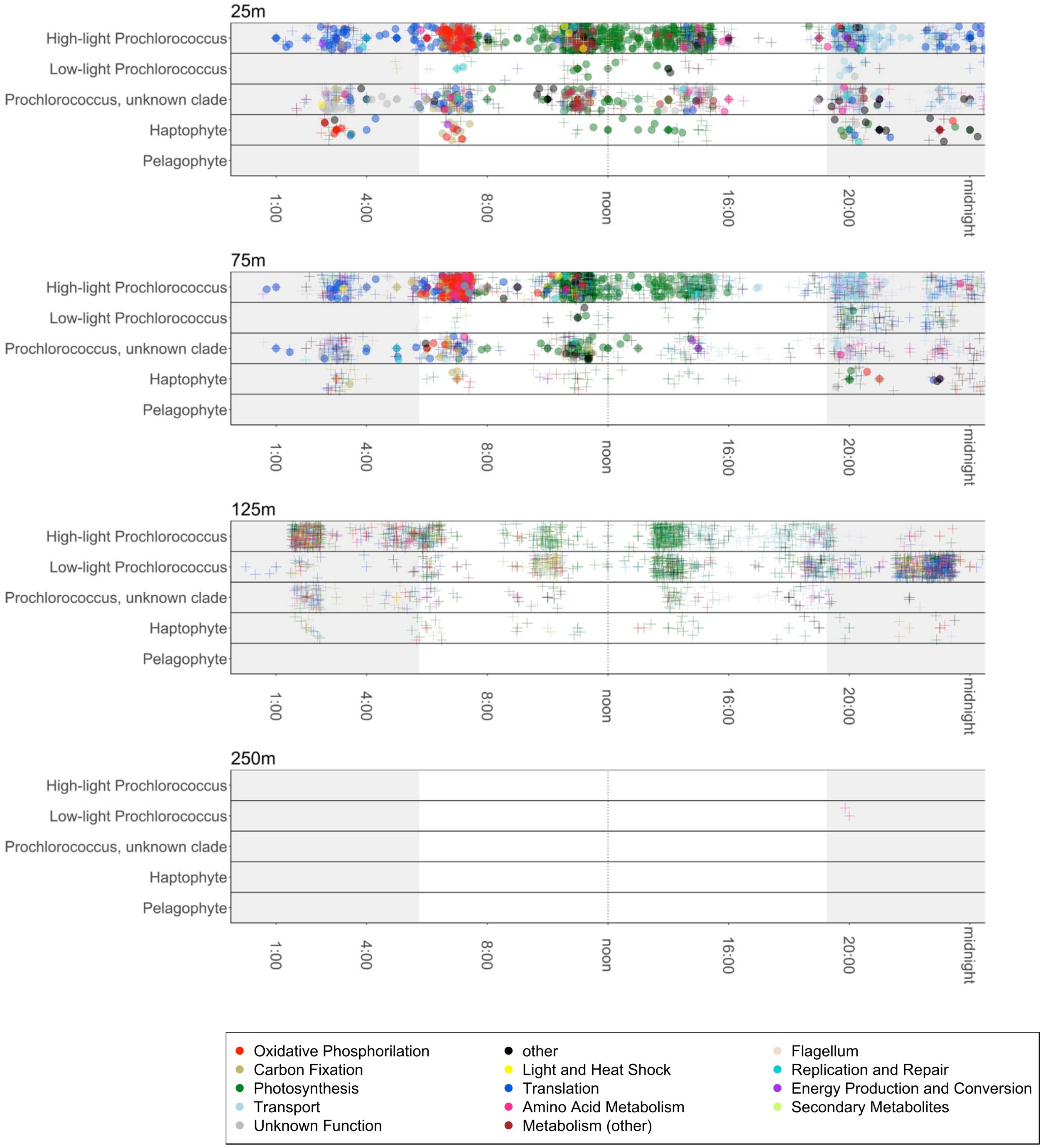

Supplementary Figure S8. Functions and peak times of all transcripts in March belonging in non-photoautotrophic microbial taxa which exhibited significant diel periodicity

Points represent peak times of gene transcript abundance with depth, by taxa (rows) and function (color) of all heterotrophic or chemolithotrophic taxonomic groups which exhibited periodicity. This figure represents the March cruise. Filled in circles represent peak times of genes expressed with significant periodicity, whereas plus signs represent peak times of genes that are not significantly periodic, according to the RAIN test.

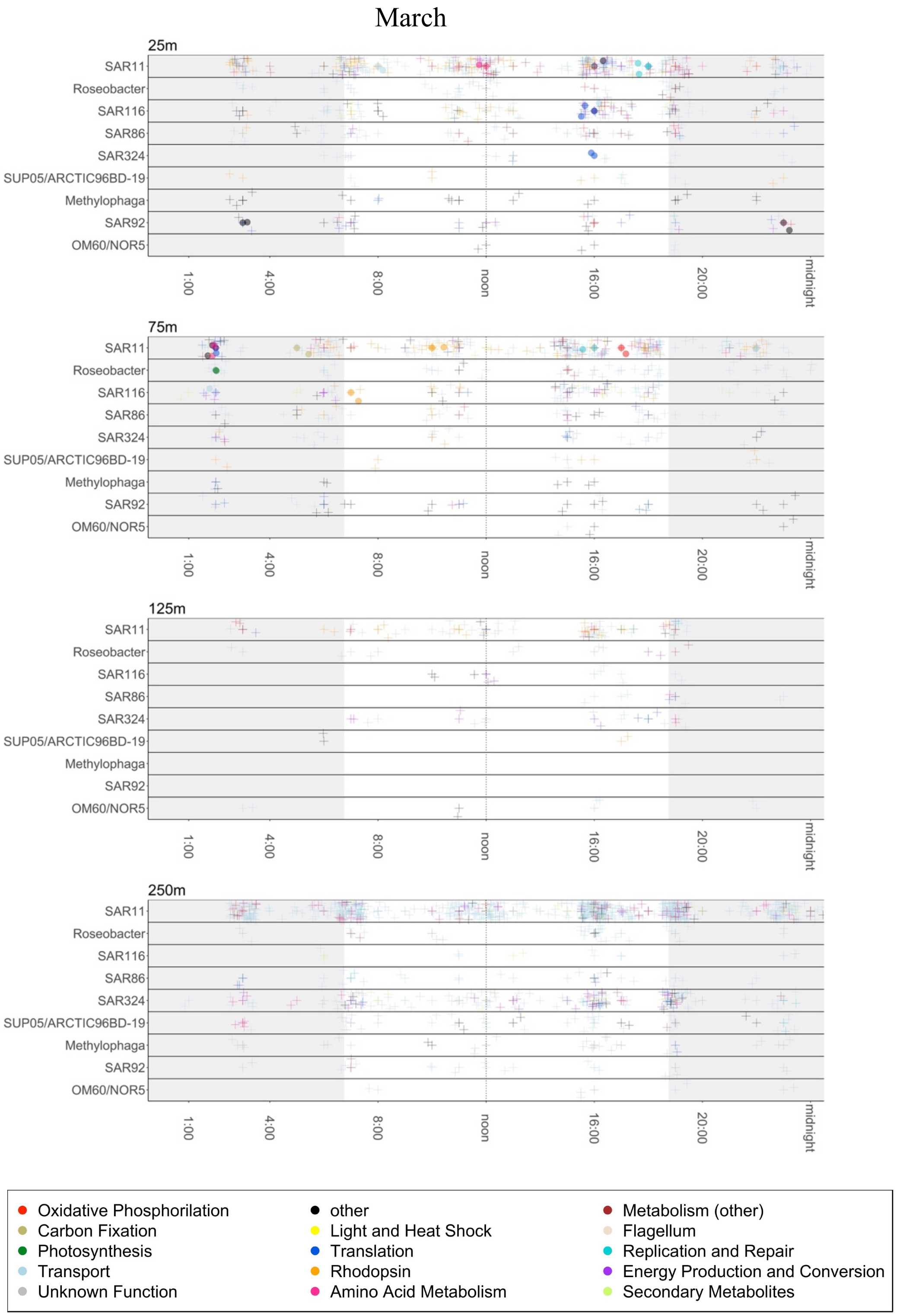

Supplementary Figure S9. Functions and peak times of all transcripts in June belonging in non-photoautotrophic microbial taxa which exhibited significant diel periodicity.

Points represent peak times of gene transcript abundance, with depth by taxa (rows) and function (color) of all heterotrophic or chemolithotrophic taxonomic groups included in the analysis which exhibited periodicity in at least one of the databases. This figure represents the June cruise. Filled in circles represent peak times of genes expressed with significant periodicity, whereas plus signs represent peak times of genes that are not significantly periodic, according to the RAIN test.

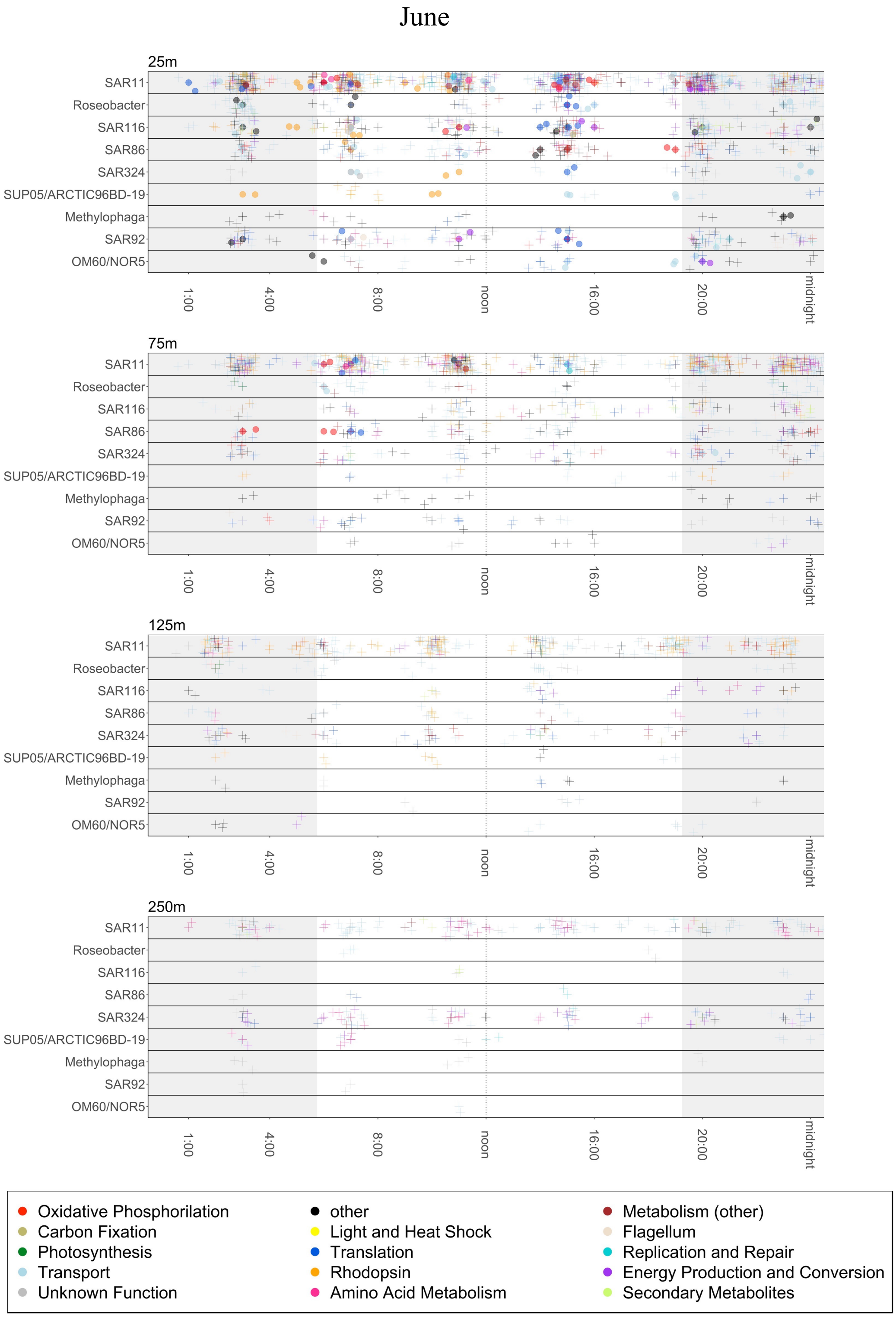

Supplementary Table 1. Average residual of gene transcript levels from the mean

For each sample, the average residual from the mean in counts of transcripts mapping to Station ALOHA reference catalog genes was found for every gene across the time series, then those averages were averaged across all genes in each sample.

	March	June
25 m	17.7	17.4
75 m	17.8	22.3
125 m	18.4	20.3
250 m	8.5	6.9
